# Interpreting ECG‐Based Risk Stratification in Women With INOCA: Methodological Considerations From the WISE Cohort

**DOI:** 10.1111/anec.70202

**Published:** 2026-07-01

**Authors:** Muhammet Cihat Çelik

**Affiliations:** ^1^ Department of Cardiology Hitit University Erol Olçok Education and Research Hospital Corum Turkey

To the Editor,

We read with great interest the recent study by Dungan et al. evaluating electrocardiographic predictors of major adverse cardiovascular events (MACE) among women with suspected ischemia and no obstructive coronary artery disease (INOCA) from the Women's Ischemia Syndrome Evaluation cohort (Dungan et al. 2026). The authors identified resting heart rate as an independent predictor of incident composite MACE and highlighted its potential role as a clinically accessible risk marker. Given the increasing recognition of INOCA as a clinically relevant condition, the study represents an important contribution to sex‐specific cardiovascular risk stratification. However, several methodological aspects deserve further clarification to better contextualize the clinical implications of these findings.

The magnitude of association between resting heart rate and MACE appears modest, with a hazard ratio close to unity despite statistical significance. Such small effect sizes may reflect residual confounding or composite endpoint sensitivity rather than a robust independent prognostic signal. Prior large‐scale cohort analyses have demonstrated that resting heart rate is associated with cardiovascular and all‐cause mortality primarily when interpreted within broader cardiometabolic and autonomic frameworks rather than as a standalone predictor (Lonn et al. 2014). Therefore, caution is warranted before translating these observations into clinical decision‐making algorithms specific to INOCA populations.

Another important consideration relates to the heterogeneity of the composite MACE endpoint used in the study. Inclusion of angina‐related hospitalization alongside hard endpoints such as myocardial infarction, stroke, and death may increase event counts but potentially reduces prognostic specificity. Previous longitudinal analyses of women with INOCA have shown that outcome interpretation varies substantially depending on endpoint composition and follow‐up duration, particularly when symptom‐driven hospitalizations are incorporated into composite cardiovascular outcomes (Jespersen et al. 2012). Stratified analyses separating hard and soft endpoints might therefore provide additional insight into the independent contribution of electrocardiographic markers.

Medication exposure immediately prior to ECG acquisition also represents a potential source of confounding. In particular, nitrate use within 24 h before ECG recording emerged as an independent predictor in the multivariable model, raising the possibility that resting heart rate may partly reflect treatment intensity or baseline symptom burden rather than intrinsic autonomic risk signaling. Previous investigations of INOCA cohorts have emphasized the complex interaction between symptom severity, therapeutic exposure, and outcome risk, suggesting that treatment‐related variables may act as surrogate markers rather than causal predictors (Mehta et al. 2022).

Finally, although resting ECG indices are attractive because of their accessibility, the absence of stress ECG parameters, heart rate variability metrics, or coronary functional testing data may have limited the mechanistic interpretation of the findings. Contemporary studies of INOCA increasingly emphasize the importance of coronary microvascular dysfunction and autonomic imbalance in determining long‐term outcomes, supporting the integration of functional and physiologic markers alongside standard electrocardiographic parameters for improved prognostic modeling (Reynolds et al. 2021). Future investigations incorporating multimodal risk assessment strategies may therefore better define the role of ECG‐derived predictors in women with INOCA.

## Author Contributions

All of the authors contributed planning, writing, and revision.

## Funding

The author has nothing to report.

## Conflicts of Interest

The author declares no conflicts of interest.

## Data Availability

Data sharing not applicable to this article as no datasets were generated or analysed during the current study.
